# Diet or Exercise Interventions vs Combined Behavioral Weight Management Programs: A Systematic Review and Meta-Analysis of Direct Comparisons

**DOI:** 10.1016/j.jand.2014.07.005

**Published:** 2014-10

**Authors:** David J. Johns, Jamie Hartmann-Boyce, Susan A. Jebb, Paul Aveyard

**Keywords:** Weight loss, Obesity, Diet, Exercise, Behavioral programme

## Abstract

Weight loss can reduce the health risks associated with being overweight or obese. However, the most effective method of weight loss remains unclear. Some programs emphasize physical activity, others diet, but existing evidence is mixed as to whether these are more effective individually or in combination. We aimed to examine the clinical effectiveness of combined behavioral weight management programs (BWMPs) targeting weight loss in comparison to single component programs, using within study comparisons. We included randomized controlled trials of combined BWMPs compared with diet-only or physical activity-only programs with at least 12 months of follow-up, conducted in overweight and obese adults (body mass index ≥25). Systematic searches of nine databases were run and two reviewers extracted data independently. Random effects meta-analyses were conducted for mean difference in weight change at 3 to 6 months and 12 to 18 months using a baseline observation carried forward approach for combined BWMPs vs diet-only BWMPs and combined BWMPs vs physical activity-only BWMPs. In total, eight studies were included, representing 1,022 participants, the majority of whom were women. Six studies met the inclusion criteria for combined BWMP vs diet-only. Pooled results showed no significant difference in weight loss from baseline or at 3 to 6 months between the BWMPs and diet-only arms (–0.62 kg; 95% CI –1.67 to 0.44). However, at 12 months, a significantly greater weight-loss was detected in the combined BWMPs (–1.72 kg; 95% CI –2.80 to –0.64). Five studies met the inclusion criteria for combined BWMP vs physical activity-only. Pooled results showed significantly greater weight loss in the combined BWMPs at 3 to 6 months (–5.33 kg; 95% CI –7.61 to –3.04) and 12 to 18 months (–6.29 kg; 95% CI –7.33 to –5.25). Weight loss is similar in the short-term for diet-only and combined BWMPs but in the longer-term weight loss is increased when diet and physical activity are combined. Programs based on physical activity alone are less effective than combined BWMPs in both the short and long term.

**Continuing Professional Education (CPE) Information**

To take the Continuing Professional Education quiz for this article, log in to www.eatright.org, click the “myAcademy” link under your name at the top of the homepage, select “Journal Quiz” from the menu on your myAcademy page, click “Journal Article Quiz” on the next page, and then click the “Additional Journal CPE Articles” button to view a list of available quizzes, from which you may select the quiz for this article.

Excess weight is a global public health issue and an important feature in discussions on the strategy for primary and secondary health care. Between 1980 and 2008, age-standardized mean global body mass index (BMI) increased by 0.4 to 0.5 per decade in men and women.^1^ Globally, in 2008, an estimated 1.46 billion adults were overweight and an estimated 205 million men and 297 million women older than age 20 years were obese.^1^ Furthermore, by 2030 estimates suggest up to 57.8% of the world's adult population (3.3 billion people) could be either overweight or obese.^2^

Substantial epidemiologic evidence suggests raised BMI is a risk factor for mortality and morbidity from a number of chronic diseases, including type 2 diabetes, cardiovascular disease, and several cancers.^3^, ^4^, ^5^ This places an economic burden on health systems and the wider economy.^6^, ^7^, ^8^ However, improvements in disease risk factors and quality of life have been observed after a modest weight loss.^9^, ^10^, ^11^ Identifying effective interventions is an important component in public health efforts to curb obesity, but the most effective strategies for weight loss are unclear.

The inclusion of diet and/or physical activity in behavioral weight management programs (BWMPs) is an important issue for health services with implications for staff training and cost. Only two previous reviews have evaluated direct comparisons between diet-only programs and those combining diet and physical activity.^12^, ^13^ One suggested that combined programs were more effective for weight loss at 12 months than diet-only programs,^12^ whereas the other found no significant differences.^13^ To our knowledge, no reviews have evaluated direct comparisons of combined programs with physical activity-only programs. Furthermore, weight-loss studies report a variety of outcomes measures, including reporting weight loss by complete cases, baseline observation carried forward (BOCF), and other intention-to-treat methods. This inconsistency in the outcome measures pooled in previous reviews makes it difficult to compare studies.

We set out to systematically review direct comparisons from randomized controlled trials in overweight and obese adults to evaluate whether BWMPs involving both diet and physical activity lead to greater weight loss at 12 months or longer than those programs involving diet only or physical activity only.

## Methods

### Search Strategy and Inclusion Criteria

A review protocol was agreed before commencing work (see Figure 1 [used with permission from the National Institute for Health and Care Excellence], available online at www.andjrnl.org). Search strategies were largely based on those used in Loveman and colleagues^14^ using the terms *diet*, *physical activity*, *weight loss interventions*, and *obese and overweight adults*. We searched BIOSIS, the Cochrane Database of Systematic Reviews, CENTRAL, the Conference Proceedings Citation Index, the Database of Abstracts of Reviews and Effects, EMBASE, the Health Technology Assessment database, MEDLINE, PsycINFO, and Science Citation Index for dates between May 2009 and November 2012 for randomized and quasirandomized controlled trials. We searched for published studies in any language. The electronic search strategy for MEDLINE is listed in Figure 2 (used with permission from the National Institute for Health and Care Excellence; available online at www.andjrnl.org). Studies predating this search were identified from Loveman and colleagues.^14^ References from relevant systematic reviews were screened and studies were also sought from experts in the field. Evidence submitted as part of a call for evidence from the UK National Institute of Health and Clinical Excellence was also examined.

Studies were included if they recruited adults (aged ≥18 years) classified as overweight or obese (people with a BMI ≥25 and ≥30, respectively, or a BMI ≥23 in Asian populations). Our focus was on weight loss interventions for the general overweight/obese population, so we excluded studies in pregnant women, people with eating disorders, and programs where the weight loss intervention was treatment for a specific medical disorder, except where those disorders were asymptomatic risk factors such as hyperlipidemia, hypertension, or prediabetes. We included trials of interventions in overweight populations where participants who had medical complications of obesity, such as a myocardial infarction, were part of a mixed population.

Interventions had to include a clearly defined BWMP that included both diet and physical activity components (ie, the intervention incorporated diet and physical activity and employed a behavior strategy with each element clearly described) and a diet- and/or physical activity-only intervention. All interventions had to involve multiple contacts. We excluded programs that involved the use of any surgery or medication, over the counter or otherwise. Interventions incorporating other lifestyle changes such as efforts at smoking cessation or reduction of alcohol intake were not included. Finally, studies were required to have a measure of weight change at 12 months or greater from baseline.

### Data Collection

Titles and abstracts were assessed by a single reviewer with a sample checked by a second reviewer. Two reviewers independently conducted data extraction and quality assessment. Any discrepancies were resolved by discussion or, where needed, by referral to a third reviewer. Where further detail on the components of an intervention or outcome measures was required, we contacted study authors and conducted web searches for additional information.

Reviewers critically appraised each included study using criteria developed by the York Centre for Reviews and Dissemination.^15^ Risk of bias was assessed on the basis of generation of the randomization sequence, concealment of allocation, selective reporting, and attrition. Studies were considered to be at low risk of bias for selective reporting where all predefined outcomes were reported and to be at low risk of attrition bias if the majority of participants (>50%) were followed-up at 12 months and if the percentage of follow-up was similar across all arms (<20% difference).

Our primary outcome of interest was mean weight change calculated using BOCF at 12 to 18 months. BOCF is an intention-to-treat analysis that makes the assumption that the weight of those who do not attend an assessment has not changed since baseline. None of the included studies reported BOCF; therefore, it was calculated using complete case data as described previously.^16^ Where reported, we also extracted data on BOCF weight change at 3 months, and on diet and physical activity measures.

### Statistical Analysis

We conducted random effects meta-analyses in Review Manager version 5.2 (2012, The Nordic Cochrane Centre). Random effects models were used because the interventions differed in the types of programs offered and populations enrolled that could lead to true between-study differences in effects. Where the estimate of variance in the meta-analysis is zero, the model is the same as a fixed effects model. We examined mean differences in weight change between intervention groups and control at 12 months and at 3 to 6 months, where reported. There were not sufficient data to meta-analyze diet and physical activity measures.

We conducted separate meta-analyses for comparisons with diet-only and physical activity-only arms. Pooled results are presented as mean differences (in kilograms) with 95% CIs, and the *I*^2^ statistic is used to present statistical heterogeneity.^17^ Sensitivity analysis was conducted excluding studies with a potential high risk of bias. Where a study contained more than one combined BWMP arm, we split the single component arm equally to avoid double counting in the pooled result. Prediction intervals were also calculated to aid interpretation of the random effects models.^18^

## Results

**Figure 3:**
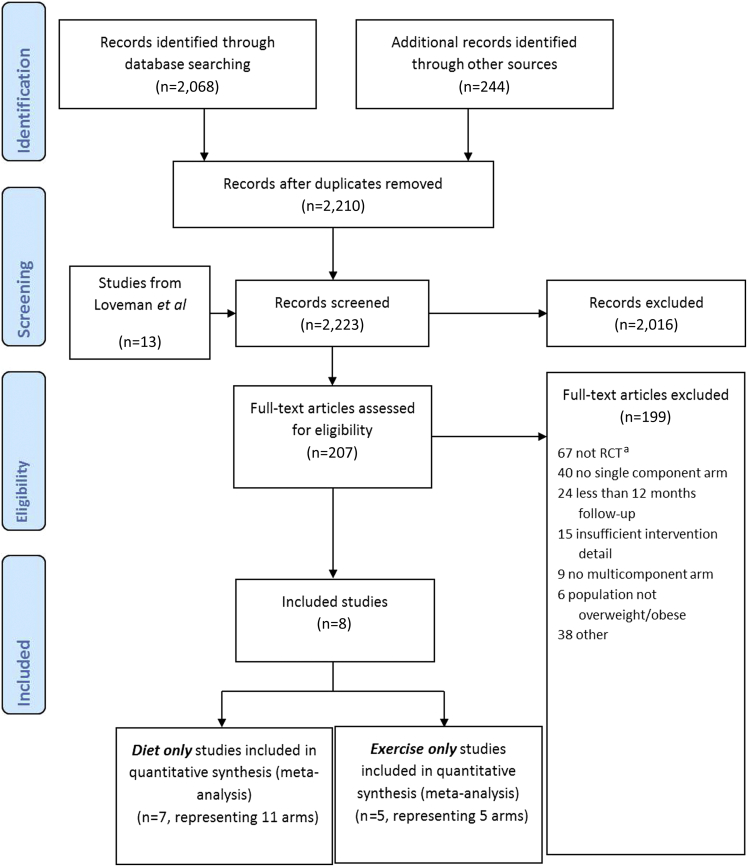
PRISMA flow diagram of review process. ^a^RCT=randomized controlled trial.

The search retrieved 2,210 references in total. Full text was retrieved and screened for 207 references. Of these, 199 were excluded, with the most common reasons being “not a randomized control trial” and a “lack of a single component arm.” Eight unique studies comparing a combined BWMP with either a diet or physical activity only intervention were identified. A flow chart detailing the search and screening process can be found in Figure 3.

### Characteristics of Included Studies

Eight studies were included, four of which included both diet-only and physical activity-only arms and, therefore, contributed to both meta-analyses. Three studies included only a comparison of diet only interventions with combined BWMPs and one study only a comparison of a physical activity only intervention with combined BWMP.

The eight included studies represented 1,022 participants. The number of participants in each study ranged from 59 to 352, with a mean of 128 participants per study. One study was conducted in Sweden, one in Belgium, and six in the United States. The mean age of study participants ranged from 32 to 70 years and, as is common in weight loss studies, the majority of participants were women (median 86%; range=49% to 100%). The mean BMI ranged from 29.2 to 37.3. Seven studies reported weight change at 12 months from baseline and one at 18 months.

All interventions were delivered in person. Of the included interventions, five were delivered by dietitians, one by professional interventionists and one by clinical psychologists. Four were, in the most part, conducted with weekly sessions; one had fortnightly sessions; one monthly; and one bimonthly. All dietary interventions included energy restriction and a recommendation to consume a low-fat diet. Physical activity interventions advised moderate to high intensity physical activity (eg, brisk walking) 3 to 5 times per week. In one intervention comparing diet only and two combined BWMPs, the combined BWMPs included supervised strength training or vibration plates as the physical activity component.^19^ Diet and physical activity components were the same in intervention and comparison arms. Further details on the characteristics of each included study can be found in Table 1.

**Table 1:** Characteristics of included studies in a systematic review and meta-analysis of direct comparisons between diet and physical activity combined behavioral weight management programs (BWMPs) and diet (D) or physical activity (PA) only BWMPs

Study	Country	Comparison	Population	Content		Delivery			Percentage followed up at 12 mo
				Diet	Physical activity	Diet component	Physical activity component	Combined BWMP	
Bertz and colleagues, 2012^22^	Sweden	BWMP vs D BWMP vs PA	N=68 Female: 100% Mean baseline BMI^a^ (SD^b^): D=30.0 (2.6); PA=30.4 (3.1); D+PA= 29.2 (2.2); Control=30.2 (3.4) Additional inclusion criteria: Women 8-12 wk postpartum	Calorie restriction (deficit of 500 kcal/d)	Brisk walking (moderate intensity), supervised twice, and recommended 4 d/wk, with length of each session incremental to 45 min	• Individual in person sessions • Delivered by dietitians • 2 sessions (1.5 h at baseline, 1 h at 6 wk)	• Delivered by registered physical therapists • 2 sessions (1.5 h at baseline, 1 h at 6 wk)	Women in D+PA^b^ group got an additional 2 h of contact, 2 sessions (2.5 h at baseline and 2 h at 6 wk)	Total: 92% BWMP: 100%, D: 76%, PA: 83%
Foster-Schubert and colleagues, 2012^20^	United States	BWMP vs D BWMP vs PA	N=439 Female: 100% Mean baseline BMI (SD): D+PA=31.0 (4.3); D=31.0 (3.9); PA=30.7 (3.7); Control=30.7 (3.9) Additional inclusion criteria: Postmenopausal women	Reduced-calorie and low-fat (1,200-2,000 kcal/d based on baseline weight)	Recommended and supervised moderate to high intensity PA, 45 min 5 d/wk	• Delivered by a dietitian with training in behavior modification • 0-24 w: 2 individual sessions and weekly group sessions (26 contacts) • 24-52 wk: at least twice monthly contact in group or by telephone (12 contacts)	• Group • Delivered by a PA physiologist • Supervised PA 3 times/wk (156 contacts)	Participants received both components; therefore, had a total of 194 contacts	Total: 91% BWMP: 92%, D: 89%, PA: 91%
Rejeski and colleagues, 2011^21^	United States	BWMP vs PA	N=288 Female: 67% Mean baseline BMI (SD): D+PA: 33.1 (4.1); PA: 32.8 (3.9); Control: 32.6 (3.5) Additional inclusion criteria: Older adults with evidence of cardiovascular disease or metabolic syndrome and self-reported mobility limitation	Reduced-calorie diet (1,200-1,500 kcal/d if baseline weight <113.4 kg, 1,500-1,800 kcal/d if ≥113.4 kg)	Recommended and supervised, moderate intensity PA, at least 5 d/wk, 30-45 min per session	• Group and individual, in person and via telephone • Delivered by a “Professional interventionists” (degree in health sciences, trained by study investigators) • 48 sessions of 10-90 min over 18 mo	• Group and individual, in person and via telephone • Delivered by a “Professional interventionists” (degree in health sciences, trained by study investigators) • 48 sessions of 10-90 min over 18 mo	No differences in length or number of contacts between PA only BWMP and D+PA BWMP	Total: 86% BWMP: 96% PA: 86%
Skender and colleagues, 1996^23^	United States	BWMP vs D BWMP vs PA	N=127 Female: 49% Mean baseline BMI: Not reported Additional inclusion criteria: Not applicable	“Controlled energy intake” diet, calories not reported, 30% fat, 50% carbohydrate, 20% protein, using Help Your Heart Eating Plan	Recommended and supervised brisk walking (“vigorous” but not “strenuous”), gradual to 45 min or more 3 to 5 times/wk	• In person, group sessions • Dietitians • 18 sessions of 60 min over 12 mo (weekly for first 12 wk, then declining in frequency)	• 3 to 5 times weekly • 208 sessions	Participants received both components therefore had a total of 226 contacts	Total: 67% BWMP: 64% D: 69% PA: 70%
Villareal and colleagues, 2011^27^	United States	BWMP vs D BWMP vs PA	N=107 Female: 63% Mean baseline BMI (SD): D+PA: 37.2 (5.4); D: 37.2 (4.5); PA only: 36.9 (5.4); Control: 37.3 (4.7) Additional inclusion criteria: Aged 65 y or older; mild to moderate frailty	Calorie restriction of 500-750 kcal/d (determined by resting energy expenditure×1.7)	Supervised activity sessions (3/wk) of 90 min, including moderate- to high- intensity PA (gradual increase to 70%-80% of peak heart rate)	• In person, group sessions • Delivered by a dietitian • Weekly sessions with a dietitian over 1 y (52 sessions)	• In person, group sessions • Delivered by a physical therapist • Three PA sessions a week for a 1 y (156 sessions)	Participants received both components therefore had a total of 208 contacts	Total: 87% BWMP: 89% D: 88% PA: 85%
Vissers and colleagues, 2010^19^	Belgium	1) BWMP (Fitness) vs D 2) BWMP (Vibration) vs D	N=79 Female: Not reported Mean baseline BMI (SD): D+vibration: 33.19 (4.7); D+fitness: 33.1 (3.4); D only: 32.9 (3.1); Control: 30.8 (3.4) Additional inclusion criteria: Not applicable	Hypocaloric diet calculated on an individual level using: (resting metabolic rate×1.3)–600 kcal/d	1) Aerobic interval training+general muscle strengthening exercise 2) Whole body vibration – exercises chosen to train all major muscle groups with machine frequency increasing from 30-35 and finally 40 Hz	• Individual, in person sessions • Delivered by a dietitian • 12 sessions over 12 mo as: • 0-3 mo: Every fortnight • 3-6 mo: Once a month • 6-12 mo: 3 more visits	• Individual sessions • Delivered by a physiotherapist 1. *Fitness* *0-3 mo:* 2 supervised and 1 home/wk *3-6 mo:* 1 supervised session and 2 home/wk *6-12 mo:* advised to maintain an active lifestyle 2. *Vibration* *0-3 mo:* Static exercises on whole body vibration platform *3-6 mo:* Dynamic exercises *6-12 mo:* Advised to maintain an active lifestyle	*BWMP Fitness* Participants received the additional 36 physical activity contacts taking their total sessions to 48 *BWMP vibration* Participants received additional contacts but number unclear	Total: 77% BWMP (fitness): 95% BWMP (vibration): 72% D: 60%
Wadden and colleagues, 1988^24^	United States	BWMP vs D (A second BWMP was not included as its diet component was not comparable to the D only arm)	N=59 Female: 86% Mean baseline BMI: Not reported Additional inclusion criteria: Not applicable	Energy-restricted diet, including very-low-energy component. Month 1 1,000-1,200 kcal/d, Months 2 and 3 400-500 kcal/d, Month 4 “refeeding,” Months 5 and 6 1,000-1,200 kcal/d	Recommended moderate PA (walking and using stairs), frequency not reported	• Group face-to-face sessions • Delivered by doctoral-level clinical psychologists • 37 sessions of 90 min each over 18 mo (weekly for first 6 mo, then declining in frequency)	• No supervised sessions	No differences in length or number of contacts between the D BWMP and D+PA BWMP	Total: 81% BWMP: 74% Diet: 83%
Wadden and colleagues, 1997^25^	United States	1) BWMP (aerobic) vs D 2) BWMP (Strength) vs D 3) BWMP (Combined aerobic and strength) vs D	N=120 Women: 100% Mean baseline BMI (SD): D+aerobic PA: 37.2 (5.1) D+strength PA: 36.5 (6) D+combined PA: 35.3 (4.4) D only: 36.4 (5.5) Additional inclusion criteria: Women only	Calorie-restricted liquid replacement diet − *Wk 2-17:* Prescribed diet of 925 kcal/d − *Wk 18-22:* Decreased liquid diet and increased consumption of conventional foods (Wk 18: 1,053 kcal/d; Wk 19: 1,150 kcal/d; Wk 20: 1,250 kcal/d) − *Wk 22 on:* Self-selected diet of 1,500 kcal/d with 12%-15% energy from protein; 55%-60% from carbohydrate, and 25%-30% from fat	1) Supervised step aerobics classes 2) Resistance training 3) Combined (60% aerobic and 40% resistance as above)	• Group face-to-face sessions • Delivered by clinical psychologist • Followed OPTIFAST program and instructed in “traditional behavioral methods of weight control” • 42 sessions of 90 min (Weekly for Wk 1-28, biweekly from Wk 29-48, and once every 3 mo thereafter)	• In person, group sessions delivered by graduate students in exercise physiology. 1) Step aerobics classes 10 cm step then those comfortable moved to 15-20 cm step at Wk 5 2) Wk 2: Exercises performed with weight that allowed them to do 10-14 repetitions Wk 3-14: Extra set for each exercise added Wk 14 on: resistance increased if able to complete 14 reps. Wk 29-48: Given help creating own resistance workouts to replace third session 3) Combination of above for all: Wk 1-28: 3 supervised sessions/wk Wk 29-48: 2 supervised sessions/wk Wk 48 on: unsupervised	Additional 5-10 min discussion on adherence to PA program	Total: 83% BWMP (aerobic): 90% BWMP (strength): 77% BWMP (Combined): 79% Diet: 83%

a BMI=body mass index.

b SD=standard deviation.

### Risk of Bias

Of the included studies, one study was judged to be at low risk of bias across all domains,^20^ two were judged high risk in one domain,^19^, ^21^ two were not judged to be at high risk but had insufficient detail to evaluate risk of bias for “allocation concealment,” and two were not judged to be at high risk but had insufficient detail to evaluate risk or bias for “allocation concealment” and “randomization procedure.” Further detail can be found in Table 2.

**Table 2:** Risk of bias judgements for studies included in a systematic review and meta-analysis of direct comparisons between diet and physical activity combined behavioral weight management programs (BWMPs) and diet or physical activity only BWMPs^a^

Study	Random sequence generation	Allocation concealment	Attrition	Selective reporting	Notes
Wadden 1988^24^	Unclear	Unclear	Low	Low	
Skender 1996^23^	Low	Unclear	Low	Low	
Wadden 1997^25^	Unclear	Unclear	Low	Low	
Vissers 2010^19^	Unclear	Unclear	High	Low	The difference in follow-up between the fitness behavioral weight management programs (95%) and the diet-only program (60%) exceeds 20%
Rejeski 2011^21^	Unclear	Unclear	Low	High	Authors measured, but did not report, weight at 12 mo
Villareal 2011^27^	Low	Unclear	Low	Low	
Bertz 2012^22^	Low	Unclear	Low	Low	
Foster-Schubert 2012^20^	Low	Low	Low	Low	

a Where ‘low’ indicates low risk of bias in that domain, ‘unclear’ indicates insufficient information with which to judge, and ‘high’ indicates high risk of bias in that domain.

### Weight Change: Combined BWMPs vs Diet-Only Interventions

**Figure 4:**
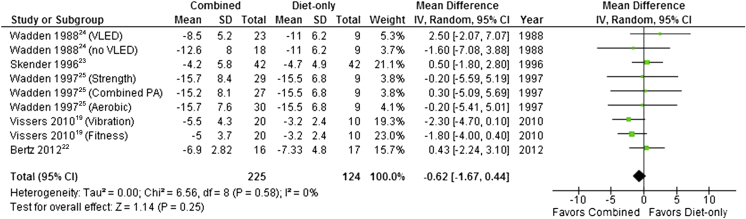
Mean difference in weight loss between behavioral weight management programs involving both diet and physical activity and programs involving diet only at 3 to 6 months. SD=standard deviation. IV=inverse variance. VLED=very-low-energy diet.

**Figure 5:**
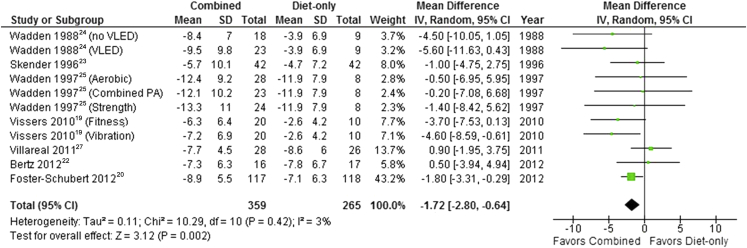
Mean difference in weight loss between behavioral weight management programs involving both diet and physical activity and programs involving diet only at 12 months. SD=standard deviation. IV=inverse variance. VLED=very-low-energy diet.

Four studies comparing a combined BWMP with a diet-only arm presented data at 3 months^19^, ^22^, ^23^, ^24^ (Figure 4) and one at 6 months.^25^, ^26^ Pooled results showed that mean weight loss at 3 to 6 months did not differ significantly between combined programs or those that included diet only (pooled mean difference –0.62 kg; 95% CI –1.67 to 0.44). Statistical heterogeneity was zero (*I*^2^=0%). However, at 12 months pooled results from 7 studies showed that mean weight loss was significantly higher in combined programs than in those that involved diet alone (mean difference=–1.72 kg, 95% CI –2.80 to –0.64) (Figure 5). Statistical heterogeneity remained low (*I*^2^=3%). A sensitivity analysis, excluding one study with high risk of bias due to attrition,^19^ produced a similar finding at both time points.

### Weight Change: Combined BWMPs vs Physical Activity–Only BWMPs

**Figure 6:**
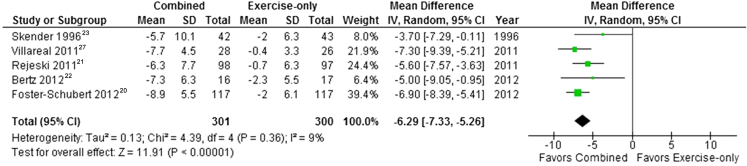
Mean difference in weight loss between behavioral weight management programs involving both diet and physical activity and programs involving physical activity only at 3 to 6 months. SD=standard deviation. IV=inverse variance.

**Figure 7:**
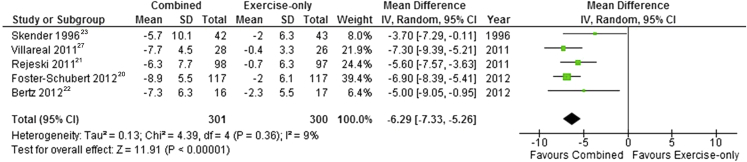
Mean difference in weight loss between behavioral weight management programs involving both diet and physical activity and programs involving physical activity only at 12 to 18 months. SD=standard deviation. IV=inverse variance.

Two studies presented data at 12 weeks^22^, ^23^ and one study at 6 months^21^ (Figure 6). Pooled results showed that weight loss at 3 to 6 months was significantly higher in combined programs than in those that involved physical activity alone (mean difference=–5.33 kg; 95% CI –7.61 to –3.04). Statistical heterogeneity was high (*I*^2^=82%). This result persisted at 12 months (mean difference=–6.29 kg; 95% CI –7.33 to –5.25; *I*^2^=9%) (Figure 7). A sensitivity analysis, excluding one study with high risk of bias^21^ from selective reporting, produced a similar finding at both time points.

### Diet and Physical Activity Measures

Only two studies reported diet variables.^20^, ^22^ They presented change in reported energy intake. Bertz and colleagues^22^ observed no difference between the combined BWMP and diet-only intervention at 3 months but observed a significantly smaller reduction in energy intake in the physical activity only intervention than in the combined BWMP. In contrast, at 12 months the combined BWMP and physical activity only intervention had a greater reduction in reported energy intake than the diet-only group. In the second study, there were no significant differences in change in reported energy intake at 12 months between the combined BWMP and the diet-only or physical activity-only arms.^20^

Four studies reported measures of physical activity: two reported step count,^20^, ^22^ one reported maximal oxygen uptake,^27^ and one measured a 400-m walking time.^21^ Two studies found no significant difference in the improvement of physical activity measures between combined BWMPs and physical activity only interventions at 12 months,^21^, ^22^ with one also finding no significant difference between diet-only and combined BWMPs. Foster Schubert and colleagues^20^ observed a significantly greater increase in steps per week in the combined BWMP than the physical activity-only or diet-only arms at 12 months. Similarly, in a study by Villareal and colleagues^27^ the combined BWMP had a greater improvement in maximal oxygen uptake than the physical activity-only and diet-only interventions.

## Discussion

Direct comparisons from randomized controlled trials show weight loss is similar in the short-term for diet-only interventions and combined BWMPs, including diet and physical activity, but in the longer-term weight-loss is greatest in combined BWMPs. Direct comparisons show that programs based on physical activity alone are less effective than combined BWMPs in both the short and long term. Programs that combine physical activity and diet lead to changes in either behavior that are at least as large as programs that focus exclusively on just one of these domains.

The summary result above relate to the average effect across the trials of combined BWMPs in comparison to either diet only or physical activity only interventions. The results provide good evidence that, on average, combined BWMPs are more beneficial than diet-only or physical activity-only interventions. However, it does not indicate whether combined BWMPs are always more beneficial. This was quantified more formally by a 95% prediction interval (PI). At 3 to 6 months (95% PI –1.95 to 0.71) PIs confirm that no difference would likely be observed between BWMP and diet-only programs in 95% of individual study settings. At 12 months (95% PI –3.17 to –0.27) there is evidence that combined BWMPs will be more beneficial than diet-only BWMPs in at least 95% of the individual study settings. Similarly, at 12 months (95% PI –8.32 to –4.26) there is also strong evidence combined BWMPs will more effective in 95% of study settings. However, at 3 to 6 months, due to low study numbers and high heterogeneity in comparison of combined BWMP and physical activity-only programs, prediction intervals cross zero (95% PI –32.8 to 22.16) and, as such, we are not able to say with confidence that our result will hold true once a greater number of studies become available.

Consistent with our findings, a systematic review of trials with direct comparisons of diet, physical activity, and behavior programs vs diet and behavior only^12^ at 12 months found a 3.02 kg (95% CI 4.94 to 1.11 kg) greater weight loss in multicomponent programs. However, a second meta-analysis (Curioni and Lourenço^13^) found no significant difference between diet-only and diet and physical activity combined programs at 12-month follow-up. None of our included studies overlapped with Avenell and colleagues,^12^ and only one of our studies^23^ overlapped with Curioni and Lourenço.^13^ This is in part due to differences in inclusion criteria because we required studies to be explicit about the physical activity and dietary interventions and we excluded trials where the whole population enrolled was using weight loss for a specific medical disorder. In addition, our review includes five studies reported after these reviews were published.

A review by Seo and Sa^28^ looked at indirect comparisons by categorizing interventions by the number of components (eg, physical activity only was one component; diet and counseling two components; and diet, physical activity, and counseling were three components). Three-component interventions had the greatest mean effect size, although time of follow-up varied greatly.

Our results indirectly suggest that the addition of diet to a physical activity intervention leads to more weight loss than the addition of physical activity to a dietary program. This hints at the relative importance of dietary change as the key component of weight-loss programs. At 6 months, diet-only interventions were as successful as BWMPs with diet and physical activity combined, although by 12 months the combined programs were superior. This suggests that although the addition of physical activity to diet may not be beneficial for initial weight loss, it may be more beneficial for maintenance of weight loss. This is consistent with the findings of studies of weight-loss maintenance.^29^

Few of our included studies reported diet and physical activity outcomes and of those that did, a variety of measures were used. There is no indication that including a dietary component hampers increases in physical activity and, in fact, some suggestion that when a program includes diet alongside physical activity, there are more favorable changes in physical activity. The evidence from measurements of dietary intake is limited but may indicate that the inclusion of a physical activity component aids long-term reductions in energy intake. This contrasts with a previous synthesis of meta-analyses and reviews that suggested, based on indirect comparisons, that although weight loss was greatest in combined diet and physical activity programs, diet-only or physical-activity only programs were most effective at improving the targeted behaviors.^30^ A greater understanding of these behaviors is needed. For instance, it has been found that the order in which diet and physical interventions are delivered within a program may influence changes in diet and physical activity.^31^

### Strengths and Limitations

Our review has several strengths. First, we looked at studies that directly compared single component and multicomponent interventions. This ensures each component was applied consistently between arms and confounding due to the mode of delivery is minimized. We calculated weight change using the BOCF method for each study to ensure consistency of effect estimates between studies and allow meta-analysis of results. We acknowledge, however, that although standardization of outcome data is a strength, BOCF itself does have limitations,^32^ especially if attrition differs significantly between arms. For example, if there is greater attrition in the single component arm, then there is the potential that the effect size will be an overestimate, assuming that the true weight change of participants who do not turn up for follow-up is significantly different from zero and different in each arm. However, only one major difference (>20%) in group-level attrition was observed in our included studies.

Publication bias is a worry for meta-analyses, especially those with a small set of included studies. Furthermore, underestimation of heterogeneity is quite common in small meta-analyses,^33^ and it is possible the standard random-effects method we used failed to detect the true heterogeneity levels. In that case our estimates would not be as conservative as they should and would be more likely to be statistically significant. However, the effects we observed at 3 to 6 and 12 to 18 months are relatively large and we would not expect our conclusions to have changed even in the presence of very high undetected study heterogeneity. One potential source of confounding is the number of contacts with program providers. Although studies maintained consistent diet and physical activity components, provision of both elements led to a greater number of contacts in the combined diet and physical activity programs in all but two studies (Table 1). It is possible that the greater accountability that may follow from greater contact frequency might have kept participants on track with physical activity and diet in the combined programs to a greater extent than occurred in the single component programs. Finally, we excluded programs where the intervention was designed to treat specific medical conditions and, as is common in weight management studies, studies contained more women than men.

## Conclusions

Our study provides important evidence that BWMPs combining diet and physical activity are more effective for weight loss over 12 months than interventions based on diet or physical activity alone. Based on the available evidence, the change observed in diet and physical activity is as large in multicomponent interventions as in single component interventions. Accordingly, practitioners can best support patients in their efforts to achieve long-term weight loss by helping them to increase physical activity and reduce energy intake within the context of BWMPs.
