# Supplementary material for: Diet or Exercise Interventions vs Combined Behavioral Weight Management Programs: A Systematic Review and Meta-Analysis of Direct Comparisons
Source: J Acad Nutr Diet. 2014 Oct;114(10):1557–68. doi: 10.1016/j.jand.2014.07.005 (PMC4180002; doi:10.1016/j.jand.2014.07.005)
Supplement: Figure 1 [file mmc1.pdf]

Supplement 1. Review protocols

Review protocol (Part a)
Managing overweight and obese adults: update review

NICE Reference	CPHE-URWMS-EV03-2012
Long title	The clinical effectiveness of long-term weight management schemes for adults: a systematic review
Project lead	Paul Aveyard (paul.aveyard@phc.ox.ac.uk)
Project manager	Jamie Hartmann-Boyce (Jamie.hartmann-boyce@phc.ox.ac.uk)
CPHE Technical Lead	Adrienne Cullum
CPHE Associate Director	Jane Huntley

Contents

Review team 23
Key deliverables and dates 24
Context 24
Purpose of this document 24
Clarification of scope 25
Review Questions 25
Outcomes 25
Inclusion criteria 26
Search methods 27
Study selection process 28
Quality assessment and data extraction 28
Data synthesis and presentation 28

Review team

This project will be conducted by a team of researchers from different institutions. The team members, and their roles on the review, will be:

Paul Aveyard, Professor of Behavioural Medicine, Department of Primary Care Health Sciences, University of Oxford	Lead systematic reviewer. Making key methodological choices within the systematic review. Chair meetings of the review team. Overall responsibility for delivery to NICE, ensuring report meets agreed protocol, discussing and agreeing with NICE any divergences from protocol. Writing and editing drafts and final report. Acting as third reviewer in cases of controversy.
Jamie Hartmann-Boyce, Research Associate, Department of Primary Care Health Sciences, University of Oxford	Systematic reviewer. Project managing the delivery of the various parts of the project. Working with NICE on search methods. Screening, appraisal and data extraction of included studies. Writing and editing drafts and final report.
David Johns, Investigator Scientist, MRC Human Nutrition Research	Systematic reviewer. Screening, appraisal and data extraction of included studies. Writing and editing drafts and final report.
Rafael Perera, Director Statistics Group, Department of Primary Health Care Sciences, University of Oxford	Statistics advice.

Note: The search will be run by Daniel Tuvey at NICE, with input from Jamie Hartmann-Boyce.

Advisory team

In addition to the core project team, we have a team of advisors who the core team will call upon the on matters relating directly to their areas of expertise, as identified below.

Carolyn Summerbell, Professor of Human Nutrition and Principal of John Snow College, Durham University	Advice on matters relating to systematic review methodology
Jane Ogden, Professor in Health Psychology, Department of Psychology, University of Surrey	Guidance on psychological theories and patients views and perceptions regarding weight loss programmes
Susan Jebb, Head of Department, Diet and Population Health, MRC Human Nutrition Research	Advice in relation to dietary prescriptions
Dawn Phillips, Public Health Portfolio Lead for Adult Obesity and Physical Activity, County Durham	Guidance on clinical aspects

Amanda Lewis, NIHR SPCR Research Fellow, Department of Primary Care Health Sciences, University of Oxford	Guidance on research into weight management in primary care
Igho Onakpoya, Researcher in Pharmacovigilance, Department of Primary Care Health Sciences, University of Oxford	Advice on systematic review methodology

Key deliverables and dates

**Deliverable**	**Date**	**Comments back from NICE CPHE by:**
1st Draft review protocol	19 October 2012	26 October 2012
Revised review protocol	30 October 2012	2 November 2012
Signing-off of review protocol	7 November 2012	
Signing-off of search strategy	5 November 2012	
Interim progress meeting/ teleconference (1) –	TBC: NICE to arrange (week of 5 November)	
Interim progress meeting/ teleconference (2) –	5 December 2013	
Draft report submitted to NICE	18 January 2013	25 January 2013
Amended report submitted to NICE	11 February 2013	
Slides for PDG meeting submitted to NICE	19 February 2013	
Review presented to PDG	26 February 2013	
Final review submitted	13 March 2013	

Context

This Review Protocol is for Review 1, with the first draft submitted by the agreed delivery date of 18 January 2013, and the final review to be submitted by 13 March 2013. A separate but related evidence review (Review 2) is covered in a separate protocol. As this is an update of an existing review (Loveman et al 2011[2]), the scope is unlikely to change beyond what is agreed here.

Purpose of this document

This document describes the aims, scope and intended methods of the update review which will be produced to support the development of NICE Public Health Guidance on lifestyle weight management programmes for overweight and obese adults.

Unless otherwise stated in this Review Protocol, this review, and its report will be conducted according to the rigorous methods described in the Cochrane Handbook, the York Centre for Reviews and Dissemination Handbook, and the 2nd Edition of the *Methods for the development of NICE public health guidance* (2009). As this is an update review it will follow

[2] Loveman E, Frampton GK, Shepher J, Picot J, Cooper K, Bryant J, et al. The clinical effectiveness and cost-effectiveness of long-term weight management schemes for adults: a systematic review. *Health Technology Assessment* 2011;15(2).

as closely as possible the scope and format of the original review (Loveman 2011) to enable direct comparison between the two, and the use of the two reviews in conjunction with one another. Where there is a discrepancy between Loveman's reporting methods and those suggested by the above listed handbooks, CPHE will be consulted.

Clarification of scope

This review aims to inform readers about the relative importance of the components included in multi-component lifestyle interventions for the treatment of obesity. This review will therefore cover only those interventions that include both a diet and exercise component, and will exclude referral to individual clinicians, management of associated conditions, surgery, and pharmacological treatments. The review will be restricted to interventions that are judged to be feasible for implementation in the UK.

For the remainder of the document, multi-component lifestyle weight management programs (LWMPs) will be defined as those which focus on reducing energy intake, increasing physical activity and changing behaviour. These may include weight management programmes, courses or clubs:

- specifically designed for adults who are obese or overweight
- that accept adults through self-referral or referral from a health practitioner
- provided by the public, private or voluntary sector
- based in the community, workplaces, primary care or online.

Review questions

The primary question in this review is similar to that of Loveman 2011, though this update will not focus on cost-effectiveness. The primary question is therefore:

- How effective and cost-effective are multi-component lifestyle weight management programmes for adults?

We will also attempt to answer secondary questions relating to these programmes. Should data be available, we will attempt to answer:

- How does effectiveness vary for different population groups (for example, men, black and minority ethnic or low-income groups)?
- How does effectiveness and cost effectiveness vary based on the components of the individual programmes (including behavioural or psychological components)?
- Are there any adverse or unintended effects associated with the use of LWMPs?

Factors which influence the effectiveness, implementation or sustainability of initiatives may be either positive ('facilitators') or negative ('barriers'), and will also be explored when assessing the included studies. However, detailed questions about key components of LWMPs, their implementation, user experience, and facilitators and barriers (overall and for specific population groups) will be addressed separately in review 2. Review 1 will focus only on the effectiveness of the LWMPs.

Outcomes

We will extract and report data on the following outcomes:

- Quantitative changes in anthropometric measures – weight, BMI, waist circumference, etc
- Intermediate measures of diet and physical activity
- Process measures such as participant satisfaction with weight management services, adherence to the intervention and attendance at sessions
- Economic outcomes (narrative only)
- Adverse effects

Inclusion criteria

For the clinical effectiveness review, we propose to follow similar criteria for including and excluding studies as used in the Loveman 2011 report, with two key changes: we will not include LWMPs that involve medications for obesity of any type, unless their use is not part of the LWMP and is comparable in both intervention and control groups; and we will include studies with 12 month follow-up or longer (Loveman required a minimum of 18 months follow-up, we will examine those studies excluded from Loveman on the basis of too short a follow-up period.. The revised inclusion criteria are listed below.

Population

- Adults (≥ 18 years) classified as overweight or obese, i.e. people with a BMI of ≥ 25 kg/m2 and ≥ 30 kg/m2, respectively.
- Studies in children, pregnant women, and people with eating disorders were not included, nor were studies specifically in people with a pre-existing medical condition such as diabetes, heart failure, uncontrolled hypertension or angina.

Intervention

- Structured, sustained multi-component weight management programmes (i.e. the intervention had to be a combination of diet and physical activity with a behaviour change strategy to influence lifestyle).
- Components of the programme had to be clearly specified (i.e. details provided of the diet, behavioural definition, and exercise components; see below).
- Programmes that included a long-term follow-up of more than 12 months.
- The programme was delivered by the health sector, in the community or commercially.
- Multi-component programmes that involved the use of any surgery or medication, OTC or otherwise, are excluded.
- Interventions incorporating other lifestyle changes such as efforts at smoking cessation or reduction of alcohol intake were not included.

Comparators

- Normal practice (as defined by the study).
- Single-component weight management strategies.
- Other structured multi-component weight management programmes.

Outcomes

- Studies were required to include a measure of weight loss.

Types of studies

- RCTs only.

- Studies published as abstracts or conference presentations were only included if sufficient details were presented to allow an appraisal of the methodology and the assessment of results to be undertaken.
- Case series, case studies, cohort studies, narrative reviews, feasibility studies, editorials and opinions were not included.
- Systematic reviews were used as a source of references.

Location

- Undertaken in any setting (i.e. community, commercial, primary care, online).
- Studies conducted in OECD countries will be considered for inclusion.[3] In the instance that a study has been conducted in an OECD country but the reviewers and advisory panel judge that the intervention would not be feasible for implementation in the UK, the reviewers will consult with CPHE regarding its inclusion.
- Studies conducted in non OECD countries will be excluded.

Cost effectiveness

As per Loveman 2011, references identified by the search strategy for the systematic review of cost-effectiveness will be considered for inclusion only if:

- They report both health service costs and effectiveness of multicomponent adult weight management programmes

OR

- Present a systematic review of such evaluations

Unlike Loveman, initially, only UK cost effectiveness studies will be included in the search, but if this results in too few studies being included, we will consult NICE to agree on a wider search being undertaken (likely all English language OECD countries).

Search methods

This is an update of an existing review and as such the existing search strategy as published in Loveman 2011 will be used. The literature search will be run by NICE with input from one reviewer (Jamie Hartmann-Boyce). Searches will be fully documented and references will be stored in a Reference Manager database.

The detailed search strategy will be agreed separately between reviewers and the CPHE's information specialist (see schedule). Any adaptations to the Loveman 2011 strategy will be confirmed with NICE and are likely to be related to increasing the specificity of the search, given the time constraints involved.

Study selection at search stage

- Studies indexed since date of last Loveman search (December 2009)
- Studies conducted in OECD countries.

[3] The original scope specified studies in the UK only. The extension to OECD countries has been agreed with NICE with the understanding that the completion of the review by stated dates is the key priority, and that the revised scope can be limited to UK only countries if the schedule so requires.

Study selection process

Assessment for inclusion will be undertaken initially at title and/or abstract level (to identify potential papers/reports for inclusion) by a single reviewer (and a sample checked by a second reviewer), and then by examination of full papers. A third reviewer will be used to help adjudicate inclusion decisions in cases of disagreement. Where the research methods used or type of initiative evaluated are not clear from the abstract, assessment will be based upon a reading of the full paper.

Quality assessment and data extraction

For the review of clinical effectiveness, we will critically appraise the literature for inclusion using a checklist based on the York CRD approach and as described in the CPHE manual.[18] However, we will modify this slightly for behavioural intervention trials and will not evaluate included studies on the basis of blinding. We will present the appraisal in tables and summarise the findings in text as described in the CPHE manual.

Data extraction will be conducted using a pre-specified data extraction form, which will be piloted by two reviewers before its use. Data extraction and quality assessment will be done independently by two reviewers, who will then compare data extraction forms. Any discrepancies will be resolved by discussion or, where needed, by referral to a third reviewer.

If deemed to be helpful for the write-up, we will reference data extracted as part of the Loveman 2011 review, but in narrative elements of the write-up we will use the data extracted by the Loveman et al rather than re-extracting this data ourselves (full, completed data extraction forms are published in the appendices of Loveman). If we conduct meta-analyses or meta-regression (see next section), we will re-extract key outcomes from the included studies in Loveman to ensure we are using the same approach to data across all studies included in the analysis.

For the review of cost-effectiveness, we will critically appraise the literature using Lovemans' *Critical appraisal checklist of economic evaluation* (table 23, page 53). Elements of this table refer to applicability to the UK; if as discussed above we do not include cost-effectiveness literature from outside the UK, we will remove these items from the checklist. All other items will remain the same.

Data synthesis and presentation, including evidence statements

We will synthesise the data in narrative form, as Loveman et al did. However, we will consider whether meta-analysis and meta-regression could be undertaken and use the baseline observation carried forward approach with standard errors calculated as described recently.[4] This is likely to be an exploratory technique rather than a definitive guide to a single underlying effect size, and such analyses will only be conducted if appropriate data is available and if time allows.

If data and time allow, we will run a meta-regression on variables of LWMPs. Meta-regression will allow us to explore whether outcomes are associated with the various characteristics of the interventions and this will prove especially useful when it comes to giving guidance on Review 2 questions. Regardless of whether a meta-regression is performed, we will categorise studies based on the following elements (taken from Jolly et al[5]):

---

[4] Kaiser KA, Affuso O, Beasley TM, Allison DB. Getting carried away: a note showing baseline observation carried forward (BOCF) results can be calculated from published complete-cases results. Int J Obes 2012; 36(6):886-889.

[5] Jolly K, Lewis A, Beach J, Denley J, Adab P, Deeks JJ et al. Comparison of range of commercial or primary care led weight reduction programmes with minimal intervention control for weight loss in obesity: Lighten Up randomised controlled trial. BMJ 2011; 343.

- Professional background of therapies
- Training of therapist
- Assessment of therapist's competence
- Fidelity checking of intervention
- Group or individual
- Duration of sessions, frequency, programme length and setting
- Content of sessions
- Weight loss goal
- Relative emphasis on diet and exercise
- Intervention theoretical background
- Predominant behavioural change techniques used

Behavioural change techniques will be assessed through the use of a pre-defined taxonomy, included as an element of the data extraction process. Each included study will be assessed against a checklist of the taxonomy, with a dichotomous yes/no option for the reviewer to indicate if the intervention included that behavioural element. The description will be obtained through the study report, and hence it should be noted that the application of the taxonomy will be limited by the depth of description provided in the report. We will use the 40-item refined taxonomy of behaviour change techniques to help people change their physical activity and healthy eating behaviours (the CALORE taxonomy) as defined by Michie et al.[6]

Where possible, we will draw weight curves for each study, mapping weight change during intervention and weight change after intervention end and seek to summarise these as appropriate.

We will group studies by the nature of the comparison, including the nature of the control group. We will note whether the control group received an active treatment that might be expected to lower weight gain or not and try to account for this in the analysis. We will also describe the nature of the intervention e.g. the calorie prescription/deficit given, the intensity of the physical activity prescription, the length of the programme, and any ongoing support offered. If possible, we will calculate the energy expenditure prescription in METs so that it will be possible to compare calorie restriction with increased calorie burning.

Data synthesis and presentation, including evidence statements, will be conducted according to the procedures outlined in the 2nd Edition of *Methods for development of NICE public health guidance 2009* where appropriate.

Key choices in how to synthesise the included evidence, or in how to develop evidence statements for this review, will be discussed with the relevant analysts at CPHE.

[6] Susan Michie, Stefanie Ashford, Falko F. Sniehotta, Stephan U. Dombrowski, Alex Bishop & David P. French (2011): A refined taxonomy of behaviour change techniques to help people change their physical activity and healthy eating behaviours: The CALO-RE taxonomy,
Psychology & Health, 26:11, 1479-1498

Review protocol (Part b & c) Managing overweight and obese adults: evidence review

NICE Reference	CPHE-URWMS-EV03-2012
Long title	The clinical effectiveness of long-term weight management schemes for adults: a systematic review
Project lead	Paul Aveyard (paul.aveyard@phc.ox.ac.uk)
Project manager	Jamie Hartmann-Boyce (Jamie.hartmann-boyce@phc.ox.ac.uk)
CPHE Technical Lead	Adrienne Cullum
CPHE Associate Director	Jane Huntley

Contents

Review team 32
Advisory team 32
Context 33
Purpose of this document 33
Clarification of scope 33
How components of behavioural weight loss programmes affect the outcome 34
What happens to the difference in weight between people treated on a behavioural weight loss programme and a control group in the longer term? 37
What interventions can maintain weight loss after the end of a behavioural weight loss programme? 37
Inclusion criteria 37
Search methods 38
Study selection process 38
Quality assessment 38
Data synthesis and presentation, including evidence statements 38
Is there evidence to support the best practice principles that NICE proposed in its 2006 guidance? 38
Principles: helping people assess their weight and decide on a realistic healthy target weight (people should usually aim to lose 5–10% of their original weight) and aiming for a maximum weekly weight loss of 0.5–1 kg/week 39
Principle: focusing on long-term lifestyle changes rather than a short-term, quick-fix approach 40

Principle: being multicomponent, addressing both diet and activity, and offering a variety of approaches....40

Principle: using a balanced, healthy-eating approach 40

Principle: recommending regular physical activity (particularly activities that can be part of daily life, such as brisk walking and gardening) and offering practical, safe advice about being more active....40

Principle: including some behaviour change techniques, such as keeping a diary and advice on how to cope with 'lapses' and 'high-risk' situations 40

Principle: recommending and/or providing ongoing support. 40

Review team

This project will be conducted by a team of researchers from two different institutions. The team members, and their roles on the review, will be:

Paul Aveyard, Professor of Behavioural Medicine, Department of Primary Care Health Sciences, University of Oxford	Lead systematic reviewer. Making key methodological choices within the systematic review. Chair meetings of the review team. Overall responsibility for delivery to NICE, ensuring report meets agreed protocol, discussing and agreeing with NICE any divergences from protocol. Writing and editing drafts and final report. Acting as third reviewer in cases of controversy.
Jamie Hartmann-Boyce, Research Associate, Department of Primary Care Health Sciences, University of Oxford	Systematic reviewer. Project managing the delivery of the various parts of the project. Working with NICE on search methods. Screening, appraisal and data extraction of included studies. Writing and editing drafts and final report.
David Johns, Investigator Scientist, MRC Human Nutrition Research	Systematic reviewer. Screening, appraisal and data extraction of included studies. Writing and editing drafts and final report.
Rafael Perera, Director Statistics Group, Department of Primary Health Care Sciences, University of Oxford	Statistics advice.

Advisory team

In addition to the core project team, we have a team of advisors who the core team will call upon for matters relating directly to their areas of expertise, as identified below.

Carolyn Summerbell, Professor of Human Nutrition and Principal of John Snow College, Durham University	Advice on matters relating to systematic review methodology
Jane Ogden, Professor in Health Psychology, Department of Psychology, University of Surrey	Guidance on psychological theories and patients views and perceptions regarding weight loss programmes
Susan Jebb, Head of Diet and Population Health, MRC Human Nutrition Research	Advice in relation to dietary prescriptions and weight management
Dawn Phillips, Public Health Portfolio Lead for Adult Obesity and Physical Activity, County Durham	Guidance on clinical aspects
Amanda Lewis, NIHR SPCR Research Fellow, Department of Primary Care Health Sciences, University of Oxford	Guidance on research into weight management in primary care
Igho Onakpoya, Researcher in Pharmacovigilance,	Systematic reviewer. Data extraction

Department of Primary Care Health Sciences, University of Oxford	of included studies.

Key deliverables and dates

**Deliverable**	**Date**	**Comments back from NICE CPHE by:**
1st Draft review protocol	15/2/13	
Revised review protocol	25/2/13	25/2/13
Signing-off of review protocol	27/2/13	
Signing-off of search strategy	n/a	
Interim progress teleconference–	6th March<br>20th March<br>4th April	
Draft report submitted to NICE ("drip feeding approach" as per review 1a)	7 March 2013 – 21 March	14 March (on components submitted 7 March)
Amended report submitted to NICE	28 March	
Slides for PDG meeting submitted to NICE	11 April	
Review presented to PDG	16 April	
Final review submitted	30 April	

Context

This Review Protocol is for Review 1b. Review 1a, which will be presented in final form on 11.2.13 in response to fulfilment of the tender for the Update Review, commissioned by NICE. There were substantial overlaps between the two reviews. In agreement with NICE, we agreed to defer some analyses for a separate review, this is Review 1b, which also incorporates some questions from the Evidence Review tender.

Purpose of this document

This document describes the aims, scope and methods of Review 1b, which will be produced to support the development of NICE Public Health Guidance on lifestyle weight management programmes for overweight and obese adults.

Unless otherwise stated in this Review Protocol, this review, and its report will be conducted according to the rigorous methods described in the Cochrane Handbook, the York Centre for Reviews and Dissemination Handbook, and the 2nd Edition of the *Methods for the development of NICE public health guidance* (2009).

Clarification of scope

The aim of this review is to examine

1. How components of behavioural weight loss programmes affect the outcome. (This is question 2 of the Evidence Review tender)

2. What happens to the difference in weight between people treated on a behavioural weight loss programme and a control group in the longer term (once the intervention has ended)? How quickly does weight increase after the end of the programme and do the characteristics of the programme affect the rate of increase in weight? (These questions are not specified in the tender but the review team think that they are important and useful).
3. What interventions can maintain weight loss after the end of a behavioural weight loss programme? (This is question 4 of the Evidence Review tender).
4. Is there evidence to support the best practice principles that NICE proposed in its 2006 guidance? (This is question 1 of the Evidence Review).

How components of behavioural weight loss programmes affect the outcome

This is phrased in the tender as "What are the most effective and cost effective behavioural or psychological components of a lifestyle weight management programme for adults – and who might best deliver them?"

The data to answer this question will come from Review 1a and a review of a further group of trials that were uncovered during the search for studies for Review 1a. The trials in Review 1a were defined as behavioural weight loss programmes that incorporated dietary and physical activity interventions versus a control group. The control interventions were rarely no intervention at all, but we included the following as unlikely to be providing much active treatment

1. No intervention at all or leaflet/s only[7]
2. Discussion/advice/counselling in one-off session +/-leaflet
3. Seeing someone more than once for discussion of something other than weight loss.
4. Seeing someone more than once for weight management, person untrained +/- leaflets

A fifth group of studies includes those that have a behavioural weight management programme that incorporates only physical activity or diet but not both, and a sixth group of studies includes behavioural programmes with both diet and physical activity components. In this review, we will appraise such papers as were found and catalogued in Review 1a and incorporate those arms of trials excluded from Review 1a that have interventions of this type.

In Review 1a we reviewed the effectiveness of 44 different interventions and we split the interventions versus control comparisons using subgroup analyses. We considered the following questions:

1. Whether the programme is delivered in groups or individually
2. The length of the programme
3. Whether the aim was weight loss or diabetes prevention
4. Whether the programme was delivered remotely, for example by Internet, or face-to-face

[7] Note that leaflets included static websites, i.e. information and advice only, not interactive weight loss programmes, which come under 5 or 6).

Principle: focusing on long-term lifestyle changes rather than a short-term, quick-fix approach

We will use data from review 1a, considering those studies that compare lifestyle weight management programmes with a diet only comparator that lasts for less than 6 months. A 6 month cut off was chosen because subgroup analysis from review 1a suggested that studies less than 6 months were not as effective as those last 6+ months.

Principle: being multicomponent, addressing both diet and activity, and offering a variety of approaches

Review 1a examines the effectiveness of multicomponent lifestyle programmes compared with no intervention. As outlined above, in Review 1b, we will examine trials of the effectiveness of diet and physical activity interventions compared with diet only and physical activity only weight loss programmes. Meta-analysis will be used to compare programmes that include both physical activity and dietary behaviour change to programmes that include only one of those elements.

Principle: using a balanced, healthy-eating approach

We will use data from review 1a, looking specifically at studies which compare BWMPs with comparator arms where no dietary advice has been given.

Principle: recommending regular physical activity (particularly activities that can be part of daily life, such as brisk walking and gardening) and offering practical, safe advice about being more active

In Review 1b we will characterise interventions by the type of physical activity that they promote. We will classify the activities in the programme as easy to incorporate or specific exercise activities and use meta-regression to examine whether there is evidence that programmes that include this kind of activity are more effective than programmes that include other forms of activity.

Principle: including some behaviour change techniques, such as keeping a diary and advice on how to cope with 'lapses' and 'high-risk' situations

By definition, all multicomponent behavioural weight management programmes include behavioural change techniques. The key question is which techniques are associated with greater effectiveness. We are investigating these as described above.

Principle: recommending and/or providing ongoing support.

The contrast with offering ongoing support is to offer one-off advice on how to lose weight. In Review 1a we investigated whether programmes in which participants were randomised to advice, usually a single session of advice by an untrained advisor, or to a programme of ongoing support. There was convincing evidence that programmes with ongoing support were more effective than one or two episodes of advice.

In addition, the trials in Review 1a randomised participants to BWMP or control, but the BWMPs varied in length trials of programmes compared long programmes to control, while others compared short programmes to control. We will use meta-regression on the studies in Review 1b to examine whether there is data that support the notion that longer support is more effective than shorter

Table 1 BCTs from the CALORE taxonomy grouped as proposed by Michie and colleagues

Technique group	Taxonomy item
Goals and planning	05- Goal setting (behaviour)<br>06- Goal setting (outcome)<br>07- Action planning<br>08- Barrier identification/problem solving<br>10- Prompt review of behavioural goals<br>11- Prompt review of outcome goals<br>20- Provide information on where and when to perform the behaviour<br>25- Agree behavioural contract<br>35- Relapse prevention/coping planning
Reward and threat	12- Prompt rewards contingent on effort or progress towards behaviour<br>13- Provide rewards contingent on successful behaviour<br>14- Shaping<br>32- Fear arousal<br>40- Stimulate anticipation of future rewards
Regulation	36- Stress management/emotional control training<br>38- Time management
Antecedents	24- Environmental restructuring
Identity	30- Prompt identification as role model/position advocate
Self-belief	18- Prompting focus on past success<br>33- Prompt self talk
Covert learning	34- Prompt use of imagery
Feedback and monitoring	16- Prompt self-monitoring of behaviour<br>17- Prompt self-monitoring of behavioural outcome<br>19- Provide feedback on performance
Social support	29- Plan social support/social change<br>37- Motivational interviewing<br>39- General communication skills training
Shaping knowledge	21- Provide instruction on how to perform the behaviour
Natural consequences	01- Provide information on consequences of behaviour in general<br>02- Provide information on consequences of behaviour to the individual<br>31- Prompt anticipated regret
Comparison of behaviour	03- Provide information about others' approval<br>04- Provide normative information about others' behaviour<br>22- Model/Demonstrate the behaviour<br>28- Facilitate social comparison
Associations	23- Teach to use prompts/cues
Repetition and substitution	09- Set graded tasks<br>15- Prompting generalisation of a target behaviour<br>26- Prompt practice

Whereas in Review 1a we used subgroup analysis to investigate differences in effectiveness, in Review 1b we will use meta-regression. Meta-regression is more powerful because it affords us the ability to examine the effects of interventions characterised in one way while accounting for other differences between programmes. However, with 40 intervention-control comparisons, it is possible to include a maximum of four predictors to avoid over-fitting the model. Therefore there is limited scope to address all differences between

programmes. Where data exist, we will use within trial data to examine some of these questions and use the totality of evidence to draw conclusions.

What happens to the difference in weight between people treated on a behavioural weight loss programme and a control group in the longer term?

This questions relates to the maintenance of weight loss achieved by behavioural weight loss programmes. The review team will report data from Review 1a that includes:

- A trajectory of weight change for all studies.
- A meta-regression to examine whether the weight trajectory after programme end depends upon the characteristics discussed above ('How components of behavioural weight loss programmes affect the outcome'). For this analysis, we will ignore the initial weight loss and will look at how weight changes that occur after the end of the programme vary among the programme types.
- A meta-analysis where possible of within study data of trials that randomised participants to longer or shorter behavioural weight loss programmes
- A meta-regression of between study data of trials that compared behavioural weight loss programmes to control and where the length of the programme varied between studies

What interventions can maintain weight loss after the end of a behavioural weight loss programme?

To answer this question we will conduct a review of reviews with the below inclusion criteria.

Inclusion criteria

Population

- Adults (≥ 18 years) initially classified as overweight or obese prior to starting a weight loss programme, i.e. people with a BMI of ≥ 25 kg/m2 and ≥ 30 kg/m2, respectively. Enrolment in a weight loss maintenance intervention implies that people who have lost weight are enrolled. We propose no restrictions on how much weight loss has been achieved prior to enrolment in a weight loss maintenance trial.
- Reviews of trials in children, pregnant women, and people with eating disorders will not be included, nor studies specifically in people with a pre-existing medical condition such as diabetes, heart failure, uncontrolled hypertension or angina.

Intervention

Any intervention aimed at maintenance of weight loss that is not pharmacotherapy or surgery

Control

Usual care or other control condition

Types of studies

A weight loss maintenance study enrols participants who have already lost weight by means other than surgery.

Reviews of randomised controlled trials, whether systematic or unsystematic, will be included. We will not include reviews of observational studies that compare the characteristics of weight loss maintainers to those who regain weight.

Location

- Undertaken in any setting
- Studies in any country will be included, though we anticipate that reviews are likely to include overwhelmingly studies conducted in OECD countries.

Search methods

The aim is to be systematic but not comprehensive and thus the searches will concentrate on specificity over sensitivity. We have already established that there are no specific MeSH terms for weight loss maintenance. Therefore our search strategy for Review 1a, which included systematic reviews, will have located such reviews. We will therefore rerun our searches for Review 1a but remove the date restriction. We will use text word searches for relevant terms, such as 'maintenance' and 'review', to find reviews of weight loss maintenance in the thousands of papers retrieved during the search for Review 1a. In addition, we will include other reviews on the topic that are referenced in the reviews that we find as a result of this search.

Study selection process

Assessment for inclusion will be undertaken initially at title and/or abstract level (to identify potential reviews for inclusion) by a single reviewer and then by examination of full papers. A second reviewer will be used to help adjudicate inclusion decisions. Where the abstract is unclear, assessment will be based upon a reading of the full paper.

Quality assessment

One reviewer will appraise reviews using the methods for appraisal of reviews described in CPHE manual. We will produce a table relating to each review and assess its quality.

Data synthesis and presentation, including evidence statements

We will extract data on the strength of evidence for particular interventions in each review and also the applicability of the evidence to the target population. We will synthesise this narratively across reviews to examine a range of interventions that affect weight loss maintenance. It is important to note that this review will exclude behavioural weight loss programmes unless such programmes have enrolled participants who have already lost weight. Randomised trials of longer versus shorter weight loss programmes are included in Review 1a.

Is there evidence to support the best practice principles that NICE proposed in its 2006 guidance?

The current best practice principles are taken from existing NICE guidance on obesity, CG43:

The best practice principles identified in NICE guidance on management of obesity are:

Primary care organisations and local authorities should recommend to patients, or consider endorsing, self-help, commercial and community weight management programmes only if they follow best practice [4] by:

- helping people assess their weight and decide on a realistic healthy target weight (people should usually aim to lose 5–10% of their original weight)
- aiming for a maximum weekly weight loss of 0.5–1 kg
- focusing on long-term lifestyle changes rather than a short-term, quick-fix approach
- being multicomponent, addressing both diet and activity, and offering a variety of approaches
- using a balanced, healthy-eating approach
- recommending regular physical activity (particularly activities that can be part of daily life, such as brisk walking and gardening) and offering practical, safe advice about being more active
- including some behaviour change techniques, such as keeping a diary and advice on how to cope with 'lapses' and 'high-risk' situations
- recommending and/or providing ongoing support.

The data to address the question of whether these principles are evidence based will be derived from the data in Review 1a, for which there is a detailed protocol. If there are no data available in the review that are relevant, we will perform a bespoke search and, depending on the data available, may also refer to other published guidelines.

Principles: helping people assess their weight and decide on a realistic healthy target weight (people should usually aim to lose 5–10% of their original weight) and aiming for a maximum weekly weight loss of 0.5–1 kg/week

For each study in Review 1a we extract whether or not a target was set and what that target was. We will use meta-regression to examine whether studies that set targets and the weight loss target is associated with greater weight loss. However, there are several caveats. First, the nature of behavioural weight loss programmes under study is that they tend not to have very extreme goals so that there may be little variation between studies. Second, there are many dimensions on which programmes might vary and it is impossible statistically to control for all such variations and many variations will not be recorded.

The main programmes that do aim for rapid weight loss are very low calorie diets (VLCDs). However, the effectiveness of setting high weight loss goals in VLCD programmes is confounded with providing meals, which is a universal feature of VLCDs. Meal replacement was a feature of only a few of the included studies in Review 1a, so assessing the effectiveness of extreme weight loss goals net of the effect of meal replacement is challenging as there are too few behavioural weight management interventions that aimed for moderate weight loss and yet which provided meals, in the way that VLCD programmes do.

We found two programmes that incorporated VLCDs in Review 1a. These were Wadden (1988), which includes very few participants, and Weinstock (1998), which also includes few participants and has no usable outcome data presented in the paper. However, for work outside the NICE review, we have systematically searched for reviews of VLCDs, which yielded a recent systematic review (Mulholland 2012). We will examine the reviews to assess whether there is evidence that the rapid weight loss typically induced by VLCDs results in weight regain. This will be a narrative synthesis .

Principle: focusing on long-term lifestyle changes rather than a short-term, quick-fix approach

We will use data from review 1a, considering those studies that compare lifestyle weight management programmes with a diet only comparator that lasts for less than 6 months. A 6 month cut off was chosen because subgroup analysis from review 1a suggested that studies less than 6 months were not as effective as those last 6+ months.

Principle: being multicomponent, addressing both diet and activity, and offering a variety of approaches

Review 1a examines the effectiveness of multicomponent lifestyle programmes compared with no intervention. As outlined above, in Review 1b, we will examine trials of the effectiveness of diet and physical activity interventions compared with diet only and physical activity only weight loss programmes. Meta-analysis will be used to compare programmes that include both physical activity and dietary behaviour change to programmes that include only one of those elements.

Principle: using a balanced, healthy-eating approach

We will use data from review 1a, looking specifically at studies which compare BWMPs with comparator arms where no dietary advice has been given.

Principle: recommending regular physical activity (particularly activities that can be part of daily life, such as brisk walking and gardening) and offering practical, safe advice about being more active

In Review 1b we will characterise interventions by the type of physical activity that they promote. We will classify the activities in the programme as easy to incorporate or specific exercise activities and use meta-regression to examine whether there is evidence that programmes that include this kind of activity are more effective than programmes that include other forms of activity.

Principle: including some behaviour change techniques, such as keeping a diary and advice on how to cope with 'lapses' and 'high-risk' situations

By definition, all multicomponent behavioural weight management programmes include behavioural change techniques. The key question is which techniques are associated with greater effectiveness. We are investigating these as described above.

Principle: recommending and/or providing ongoing support.

The contrast with offering ongoing support is to offer one-off advice on how to lose weight. In Review 1a we investigated whether programmes in which participants were randomised to advice, usually a single session of advice by an untrained advisor, or to a programme of ongoing support. There was convincing evidence that programmes with ongoing support were more effective than one or two episodes of advice.

In addition, the trials in Review 1a randomised participants to BWMP or control, but the BWMPs varied in length trials of programmes compared long programmes to control, while others compared short programmes to control. We will use meta-regression on the studies in Review 1b to examine whether there is data that support the notion that longer support is more effective than shorter

support. We will also use meta-analysis and meta-regression to compare the effectiveness of programmes in which contact frequency or intensity declined over time (for example, initially in person sessions but then phone sessions, or initially weekly declining to monthly to trials where the intervention was of consistent intensity and ended abruptly. These data will be derived from taxonomy item 27 – use of follow-up prompts).

References

Abraham, C. & Michie, S. 2008, "A Taxonomy of Behavior Change Techniques Used in Interventions", Health Psychology, vol. 27, no. 3, pp. 379-387.

Michie, S., Ashford, S., Sniehotta, F. F., Dombrowski, S. U., Bishop, A., & French, D. P. 2011, "A refined taxonomy of behaviour change techniques to help people change their physical activity and healthy eating behaviours: The CALO-RE taxonomy", Psychology & Health, vol. 26, no. 11, pp. 1479-1498
