# Supplementary material for: Diet or Exercise Interventions vs Combined Behavioral Weight Management Programs: A Systematic Review and Meta-Analysis of Direct Comparisons
Source: J Acad Nutr Diet. 2014 Oct;114(10):1557–68. doi: 10.1016/j.jand.2014.07.005 (PMC4180002; doi:10.1016/j.jand.2014.07.005)
Supplement: Figure 2 [file mmc2.pdf]

Supplement 2. MEDLINE search strategy

Database: **Ovid MEDLINE(R)** 1946 to November Week 1 2012 (searched 07 November 2012)

Strategy used:

1	Obesity/ or Obesity, Morbid/ or Obesity, Abdominal/
2	exp weight gain/
3	Overweight/
4	(overweight or over weight or overeat* or over eat* or overfeed* or over feed*).ti,ab.
5	(weight adj1 gain*).ti,ab.
6	obes*.ti,ab.
7	or/1-6
8	(modific* or therap* or intervention* or strateg* or program* or management or scheme* or group* or pathway*).ti,ab.
9	(weight adj1 los*).ti,ab.
10	(weight adj1 reduc*).ti,ab.
11	exp weight loss/
12	8 and (9 or 10 or 11)
13	Obesity/dh, pc, th
14	Obesity, Morbid/pc, dh, th
15	8 and (13 or 14)
16	Diet Therapy/
17	Diet, Fat-Restricted/
18	Diet, Reducing/
19	Dietetics/ed, mt
20	(diet or diets or dieting).ti,ab.
21	(low calorie or hypocaloric or calorie control*).ti,ab.
22	(health* adj1 eating).ti,ab.
23	(diet* adj2 (modific* or therapy or intervention* or strateg* or program* or management or scheme*)).ti,ab.
24	(nutrition adj2 (modific* or therapy or intervention* or strateg* or program* or management or scheme*)).ti,ab.
25	(Weight Watchers or weightwatchers).ti,ab.
26	(slimming world or slimmingworld).ti,ab.
27	(lighterlife or "lighter life").ti,ab.
28	or/16-27
29	8 and 28
30	exp exercise/
31	exercise therapy/
32	(exercise and (therapy or therapies or activity or activities or class* or program* or group* or session* or scheme*)).ti,ab.

33	(Gym and (trainer* or therap* or activit* or class* or program* or group* or session* or scheme* or club*)).ti,ab.
34	(walk* or step* or jog* or run*).ti,ab.
35	(aerobic* or physical therap* or physical activit*).ti,ab.
36	(fitness adj (class or regime* or program* or group* or session* or scheme*)).ti,ab.
37	(reduc* adj2 sedentary behavio?r).ti,ab.
38	(dance and (therap* or activit* or class* or program* or group* or session* or scheme*)).ti,ab.
39	personal trainer*.ti,ab.
40	(gym or gyms or gymnasium*).ti,ab.
41	or/30-40
42	8 and (30 or 31 or 34 or 35)
43	32 or 33 or 36 or 37 or 38 or 39 or 40 or 42
44	cognitive therapy/
45	Counseling/
46	behavior therapy/
47	cognitive therapy/
48	behavio?ral intervention*.ti,ab.
49	(change* adj2 lifestyle*).ti,ab.
50	(changing adj2 lifestyle*).ti,ab.
51	(lifestyle adj2 modif*).ti,ab.
52	Hypnosis/
53	Counseling/
54	(counseling or counselling).ti,ab.
55	or/44-54
56	Randomised Controlled Trials as Topic/
57	randomised controlled trial.pt.
58	controlled clinical trial.pt.
59	Controlled Clinical Trial/
60	placebos/
61	random allocation/
62	Double-Blind Method/
63	Single-Blind Method/
64	(random* adj2 allocat*).tw.
65	placebo*.tw.
66	((singl* or doubl* or trebl* or tripl*) adj (blind* or mask*)).tw.
67	Research Design/
68	((random* or control*) adj5 (trial* or stud*)).tw.
69	Clinical Trials as Topic/
70	randomly.ab.
71	(randomised or randomized).ab.

72	Evaluation studies as topic/
73	comparative study/
74	(matched communities or matched populations).mp.
75	(control* adj (trial* or stud* or evaluation*)).mp.
76	(comparison group* or control* group*).mp.
77	Matched-Pair Analysis/
78	matched pair*.ti,ab.
79	Meta-Analysis/
80	meta analy*.ti,ab.
81	"Outcome Assessment (Health Care)"/
82	outcome stud*.ti,ab.
83	intervention studies/
84	follow up studies/
85	(systematic* adj (review* or methodolog* or research* or search*)).ti,ab.
86	((hand or manual or computer or electronic or database) and search*).ti,ab.
87	(hand adj search*).ti,ab.
88	(medline or embase or Cochrane or cinahl or psychlit or psychinfo or scisearch or pubmed).ab.
89	Health technology assessment*.ab,in.
90	(pooled adj analys*).ti,ab.
91	(electronic* adj search*).ti,ab.
92	(synthes* adj5 (literature* or research* or studies or data)).ti,ab.
93	or/56-92
94	12 or 15
95	7 and 93 and 94
96	7 and 28 and 93
97	7 and 29 and 93
98	7 and 41 and 93
99	7 and 43 and 93
100	7 and 55 and 93
101	96 or 98 or 100
102	97 or 99 or 100
103	96 and 98 and 100
104	96 and 98
105	96 and 100
106	98 and 100
107	104 or 105 or 106
108	97 and 99
109	97 and 100
110	99 and 100
111	108 or 109 or 110

112	103 or 107 or 111
113	Anti-Obesity Agents/
114	(sibutramine or orlistat or rimonabant).ti,ab,nm.
115	exp Bariatric Surgery/
116	exp obesity/su
117	113 or 114 or 115 or 116
118	112 not 117
119	limit 118 to (english language and humans)
120	limit 119 to ("all infant (birth to 23 months)" or "all child (0 to 18 years)" or "newborn infant (birth to 1 month)" or "infant (1 to 23 months)" or "preschool child (2 to 5 years)" or "child (6 to 12 years)")
121	119 not 120
122	(editorial or comment or letter).pt.
123	121 not 122
124	limit 123 to ed=20091208-20120530
**125**	**limit 123 to ed=20091208-20121031**
